# Radiation‐induced sarcomas following childhood cancer – A Canadian Sarcoma Research and Clinical Collaboration Study (CanSaRCC)

**DOI:** 10.1002/cnr2.1834

**Published:** 2023-05-13

**Authors:** Marina Parisi Dutra, Caroline Mary Rodrigues, Hagit Peretz‐Soroka, Mauricio Ribeiro, David Shultz, David Hodgson, Derek S. Tsang, Abha A. Gupta

**Affiliations:** ^1^ Division of Haematology/Oncology The Hospital for Sick Children Toronto Ontario Canada; ^2^ Division of Medical Oncology, Princess Margaret Cancer Centre University of Toronto Toronto Ontario Canada; ^3^ Canadian Sarcoma Research and Clinical Collaboration, CanSaRCC Toronto Ontario Canada; ^4^ Department of Radiation Oncology, Princess Margaret Cancer Centre University of Toronto Toronto Ontario Canada

**Keywords:** cancer survival, childhood cancer, radiation‐induced sarcoma, radiotherapy, secondary neoplasia

## Abstract

**Background:**

Radiation‐induced sarcoma (RIS) is a late toxicity of radiation therapy (RT) usually associated with poor prognosis. Due to ongoing improvements in childhood cancer treatment and patient outcomes, RIS may become more prevalent notwithstanding evolving indications for RT. Due to limited reported studies, we sought to review our experience with RIS in survivors of pediatric cancer.

**Methodology:**

Data were collected on RIS patients following treatment for childhood cancer (initial diagnosis <18 years) identified in the CanSaRCC database. Additionally, details on the protocol guidance at time of treatment were compared with current guidelines for the same disease.

**Results:**

Among 12 RIS identified, median age at initial diagnosis was 3.5 years (range 0.16–14) and the latency from RT to RIS diagnosis was 24.5 (range 5.4–46.2) years. Initial diagnoses included neuroblastoma, rhabdomyosarcoma, Ewing sarcoma, Wilms tumor, retinoblastoma and Hodgkin's Lymphoma. RIS histologies included osteosarcoma and soft tissue sarcomas. In comparison to protocols followed at time of diagnosis to current ones (2022), 7/12 (58%) patients would have required RT. RIS treatment included chemotherapy, radiation and surgery in 3/11 (27%), 10/11 (90%), and 7/11 (63%) patients, respectively. With a median follow‐up time of 4.7 years from diagnosis of RIS, 8 (66%) patients were alive and 4 (33%) had died of progressive RIS.

**Conclusion:**

RIS is a serious late effect of radiotherapy in childhood cancer; however, radiation remains an integral component of primary tumor management and requires participation from a specialized multi‐disciplinary team, aiming to mitigate RIS and other potential late effects.

## 1 INTRODUCTION

In recent decades, the treatment of childhood cancer has greatly advanced with combined modality treatment and improved supportive care, increasing the survival rates up to 80%.^1^, ^2^ As the survivorship population grows in number and time, there is a proportional increase in the cumulative risk of late effects including second neoplasia that can be up to 14% higher compared with the general population.^3^ The etiology of secondary neoplasia is usually multifactorial. Contributing factors include genetic predisposition syndromes, chemotherapy treatment and radiotherapy (RT), with an increase in risk associated with age at primary treatment, stage of primary tumor and intensity of therapy.^1^, ^4^

The risk of developing a RT associated neoplasia is reported to be 3.2%–6.4% at 20 year post primary treatment, the most common secondary diagnoses including breast, bone and thyroid cancers.^1^, ^5^ The modality of RT, RT dose and volume, and type of exposed tissues are factors that can affect the chance of developing a new neoplasia.^1^, ^2^, ^3^

Radiation‐induced sarcomas (RIS) have an incidence of 0.03%–0.2% at 10 years and can be up to 40% higher than the general population.^6^ The majority of pathological diagnosis include poorly differentiated and high‐grade tumors^6^, ^7^ however the diagnostic criteria have evolved over time, the most recent proposed by Huvos et al. in 1985, a modified criterion of Cahan et al.^8^ These criteria consider the new tumor occurring within the RT volume, a latency of years from primary tumor, previous tumor of different histological diagnosis and histological evidence of sarcoma.^8^

Even though there is a well‐established relationship between RT in the pediatric patient and the development of sarcoma secondary to treatment, there are a paucity of studies considering this specific population with the majority of RIS data based on adult exposed patients, especially breast cancer survivors. We performed an analysis of RIS in patients who received RT as children to better understand whether decision making regarding the indications for RT have evolved over time.

## 2 METHODS

### 2.1 Patients

Patients with a diagnosis of RIS between January 2005 and January 2023, history of pediatric cancer (primary cancer diagnosis <18 years of age) who received RT as part of treatment identified in the Canadian Sarcoma Clinical and Research Collaboration (CanSaRCC) database were included. Ethics approval was obtained from the Hospital for Sick Children alongside the CanSaRCC consortium agreement.

### 2.2 Data collection and analysis

Data of the primary pediatric cancer were retrospectively collected from electronic and paper medical records including demographics, treatment, RT and outcome details. Information regarding the RIS was extracted from the CanSaRCC database.

Data were presented using descriptive summary statistics. Continuous data such as age or interval diagnosis were presented as medians.

## 3 RESULTS

### 3.1 Characteristics of primary tumor

Of 12 patients identified, 9 (75%) were female. Median age at diagnosis of primary cancer was 3.5 years (range 0.5–14) and of receiving RT was 3.8 years (range 0.7–14.4). RT dose ranged from 15 to 50.4 Gy (Table 1). Of the 12 patients with available genetic information, germline features were as follows: 2 germline p53 (Li Fraumeni), 1 Gorlin syndrome, 1 germline RB mutation and 1 patient with hemihypertrophy but without confirmatory genetic testing.

**TABLE 1:** Diagnosis and demographics of pediatric RIS patients.

#	Sex	Age at diagnosis (y)	Age at RT (y)	RT dose	Primary diagnosis	Time to RIS from initial RT (y)	RIS histology	Genetics
1	F	1	1.2	15 Gy	NB stage IV	46.2	LMS	
2	F	9	9.7	45 Gy	RMS	16.6	OST	
3	F	2	3.5	50.4 Gy	ERMS parameningeal (stage III)	15	OST	P53
4	F	3	2.5	45 Gy	ERMS posterior cervical region	29.4	OST	P53
5	M	5	4.2	36 Gy + boost 18 Gy	Medulloblastoma average risk	7.4	MPNST	Gorlin
6	F	9	9.8	47.5 Gy	Hodgkin Lymphoma	40	LPS	
7	M	4	4.8	25 Gy	Wilms tumor stage I	42.5	SFT	
8	F	2	2.4	30 Gy	NB	35.6	MPNST	
9	F	0.5	0.7	Unknown	Retinoblastoma	20.5	OST	Rb
10	F	14	14.4	15 Gy + boost 10 Gy	Hodgkin Lymphoma stage IIIB	13	NA	
11	M	14	14.3	50.4 Gy	Ewing sarcoma L chest wall non metastatic	5.3	OST	
12	F	3	3.2	20 Gy	Wilms tumor stage V (with favorable histology)	40.6	LMS	Hemi‐hypertrophy

Abbreviations: ERMS, embryonal rhabdomyosarcoma; LMS, leiomyosarcoma; LPS, liposarcoma; MPNST, malignant peripheral nerve sheet tumor; NA, not available; NB, neuroblastoma; OST, osteosarcoma; SFT, solitary fibrous tumor.

### 3.2 RIS diagnosis, management and outcome

Median latency from RT to RIS diagnosis was 24.5 years (range 5.3–46.2). RIS cases were equally divided in bone sarcomas and soft tissue sarcomas (Table 1). One patient did not receive treatment, 2 were treated with only surgery, 1 with chemotherapy and surgery, 5 with RT and surgery and 2 received all three treatment modalities. One patient was currently on treatment with chemotherapy at the time of analysis. At last follow‐up (median 4.7 years, range 0–8.7), 4 died due to disease (33%), 1 alive with disease, and 6 are alive with no evidence of disease (Table 2).

**TABLE 2:** Treatment and outcome of RIS.

Patient	Chemotherapy	Surgery for RIS	RDT for RIS	Last follow up	Outcome of RIS
1	No	Yes	No	2.8	NED
2	No	Yes	No	3.2	DOD
3	Yes	Yes	No	5	NED
4	No	Yes	Yes	3.6	DOD
5	Yes	Yes	Yes	8.7	AWD
6	Yes	Yes	Yes	4.8	DOD
7	No	Yes	Yes	5.7	NED
8	No	Yes	Yes	4.7	NED
9	No	Yes	Yes	1.8	NED
10	No	Yes	Yes	6	NED
11	No	No	No	0.2	DOD
12^a^	Yes	No	No		

Abbreviations: AWD, alive with disease; DOD, died of disease; NED, no evidence of disease.

^a^ Patient on treatment at the time of this publication.

### 3.3 Prescription of RT—change over time

One patient received Intensity‐Modulated Radiation Therapy (IMRT); all other patients were treated with conventional field‐based radiation therapy. Three patients receive whole abdomen RT, 1 patient receive craniospinal RT with posterior fossa boost, and 8 patients received RT localized to the tumor field. RT doses ranged from 15 to 54 Gy.

The prescription of RT as per the protocol used at time of diagnosis was compared with the prescription of RT offered in trials in current day are compared for each patient in Table 3. Current protocols may include additional tumor genetics that were not done at time of additional diagnosis. Notwithstanding this, there are at least 40% of cases where current protocols would have omitted RT.

**TABLE 3:** Comparison of RT as per COG protocols (or similar) at primary diagnosis year and 2022.

#	Primary diagnosis (y)	Protocol at diagnosis time	RT dose by protocol	Current COG protocol (2022)	RT dose at current protocol	Change in RT over time? Y versus N
1	1974	Stage IV: CCG331	RT dose not determined by protocol	HR: ANBL1531	21.6 Gy	Y
2	1999	D9803	36–50.4 Gy	ARST1431	36–59.4 Gy	
3	2001	D9803	36–50.4 Gy	ARST1431	36–59.4 Gy	
4	1984	IRSII	45–55 Gy	ARST1431	36–59.4 Gy	
5	1989	A9961	23.4 Gy + boost − total 55.8 Gy	ACNS0331	23.4 Gy + boost − total 55.8 Gy	
6	1973	MOPP#4	36–40 Gy	AHOD1331	21–30 Gy	Y
7	1973	NWTS 2	Group 1 no RT	AREN0532	No RT	
8	1982	Stage I‐III: CCG351	25–34 Gy	1. Non‐HR: ANBL1232	1. No RT	Y
9	1990	CHP‐571	42–46 Gy	ARET2121	No RT	Y
10	2001	P9425	21 Gy	AHOD1331	21–30 Gy	
11	2012	AEWS0031	45–56.4 Gy	AEWS1031	36–55.8 Gy	
12	1982	NWTS3	20 Gy	AREN0534	No RT	Y

## 4 DISCUSSION

Radiation‐induced sarcomas are a rare late side effect of childhood cancer with associated risk factors including dose of RT, younger age at diagnosis, time since RT exposure and genetic susceptibility.^7^, ^9^ In our cohort 65% of the patients received a dose higher than 30 Gy, similar to the dose reported as higher risk in previous studies.^2^ The median time from RT to RIS diagnosis was longer (24.5 years) in our small series compared with prior reports (median 8 years) especially when considering that most of our patients also received chemotherapy as part of the treatment.^4^, ^7^ Upon review of protocols used at time of original diagnosis of the pediatric cancer compared with current therapeutic strategies, in general, the RT requirement has decreased. This has been a common trend even in adult cancers such as Hodgkin Lymphoma where RT has been treated with more aggressive/multi‐agent chemotherapy.^10^, ^11^

Genetic predisposition syndromes are associated with increased risk of development of RIS, when compared with patients without this predisposition.^1^, ^5^, ^12^ p53 (Li‐Fraumeni) and RB mutation are the most common mutations associated with RIS.^5^, ^12^ Our cohort included patients with Li‐Fraumeni, Gorlin syndrome, Rb and one with a clinical diagnosis of hemihypertrophy, without confirmatory genetic testing.

The treatment of RIS can be challenging, usually requiring multimodal therapy including surgery and more RT in order to optimize chance of cure^6^; RIS can be therefore be associated with high morbidity and mortality.

Pediatric cancer patients requiring RT should always be discussed at multi‐disciplinary tumor boards highlighting the data to support the use of RT in each case. As shown by our small series, RT is mandatory to cure some pediatric cancers despite evolution and further insights into disease biology and/or more intensive systemic therapy options. Cure of a primary neoplasm should not be compromised by fear of second malignancies or other late effects; however, we propose that oncology teams should continue to strive for precision in the indication, dose and field of RT in the treatment of childhood cancer.^13^, ^14^ Proton therapy is an emerging radiotherapeutic approach to decrease the volume of tissue being treated, and may decrease the risk of developing a secondary neoplasm; however, impact on risk of RIS remains understudied and requires longer follow‐up.^13^, ^15^, ^16^

## 5 CONCLUSION

The treatment of childhood cancer should aim for a prolonged overall survival associated with a decrease in long term side effects. In order to decrease the chances of RIS, the indication of RT as well as dosage, fields and radiotherapy techniques should be considered carefully to maximize the chance of cure while minimizing long‐term toxicities, and the treatment of childhood cancer should be carried out by multidisciplinary teams.

## AUTHOR CONTRIBUTIONS

**Marina Parisi Dutra:** Conceptualization (equal); data curation (equal); formal analysis (equal); methodology (equal); writing – original draft (equal). **Caroline Mary Rodrigues:** data curation (equal). **Hagit Peretz‐Soroka:** Conceptualization (equal); data curation (equal); formal analysis (equal); writing – review and editing (equal). **Mauricio Ribeiro:** Writing – review and editing (equal). **David Shultz:** Writing – review and editing (equal). **David Hodgson:** Writing – review and editing (equal). **Derek S. Tsang:** Writing – review and editing (equal). **Abha A. Gupta**: Conceptualization (equal); data curation (equal); formal analysis (equal); methodology (equal); writing – original draft (equal).

## CONFLICT OF INTEREST STATEMENT

DST received travel funding from Mevion Medical Systems and Elekta AB in 2022. Otherwise, the authors certify that they have NO affiliations with or involvement in any organization or entity with any financial interest, or non‐financial interest in the subject matter or materials discussed in this manuscript.

## Data Availability

Data sharing is not applicable to this article as no new data were created or analyzed in this study.
